# Caregivers' experiences and perceptions of suicidality among their children and youth with fetal alcohol spectrum disorder

**DOI:** 10.3389/fpsyt.2022.931528

**Published:** 2022-08-31

**Authors:** Kelly D. Harding, Kailyn Turner, Stephanie J. Howe, Mercedes Jayne Bagshawe, Katherine Flannigan, Mansfield Mela, Carly A. McMorris, Dorothy Badry

**Affiliations:** ^1^Canada Fetal Alcohol Spectrum Disorder Research Network, Vancouver, BC, Canada; ^2^Department of Psychology, Laurentian University, Sudbury, ON, Canada; ^3^Werklund School of Education, University of Calgary, Calgary, AB, Canada; ^4^Alberta Children's Hospital Research Institute, Calgary, AB, Canada; ^5^Department of Psychiatry, University of Saskatchewan, Saskatoon, SK, Canada; ^6^Faculty of Social Work, University of Calgary, Calgary, AB, Canada

**Keywords:** fetal alcohol spectrum disorder, caregivers', lived experience, suicide, mental health, suicidality, social-ecological model

## Abstract

Individuals with Fetal Alcohol Spectrum Disorder (FASD) experience a range of biopsychosocial vulnerabilities that can increase the possibility of adverse life outcomes, including a heightened risk of suicidality. In this study, we explored the lived experiences of caregivers of children and youth with FASD and suicidality, including their perceptions of their child and youth's suicidal experiences. Between March and June 2021, six comprehensive, semi-structured interviews were conducted with five caregivers of children and youth with FASD (*M*age = 14.5 years, range 11–22) who were currently experiencing suicidality or had a history of suicidality. Data were analyzed using interpretative phenomenological analysis and then developed into a composite vignette informed and organized by the social-ecological suicide prevention model (SESPM). The composite vignette revealed the narratives of families living with and caring for children and youth with FASD who experience suicidality in relation to the complex and intersectional individual, relational, community, and societal level contextual and protective factors. Findings from this study highlight the critical need for comprehensive FASD-informed suicide prevention and intervention approaches to promote the mental health and wellbeing of children and youth with FASD and their caregivers.

## Introduction

Fetal alcohol spectrum disorder (FASD) is a neurodevelopmental disorder caused by prenatal exposure to alcohol and is estimated to impact at least 4–7% of the North American population (1–4). Individuals with FASD experience multifaceted brain- and body-based difficulties, ranging from physical health challenges to impairments in cognitive, behavioral, social-emotional, and adaptive functioning (5). Related to these challenges, and in the absence of adequate support, individuals with FASD may also experience complex life adversity such as school disruption, difficulties obtaining and maintaining employment, financial and housing instability, trouble with the law, and mental health and substance use concerns and disorders (6, 7). Further compounding these biopsychosocial vulnerabilities, experiences of trauma and victimization are exceptionally common for individuals with FASD (8, 9). Concerningly, many of the complex challenges experienced by individuals with FASD (e.g., trauma, mental health and substance use issues) overlap with risk factors for suicidality, and there is growing evidence of elevated risk for suicidal ideation and behavior in this population (10).

### Suicidality and FASD

Several decades ago, researchers reported that individuals with FASD experience remarkably high rates of suicidality (11). Depending on the stage of suicidality (i.e., ideation, attempt, or death), time frame (i.e., lifetime or current), and context or setting investigated (e.g., child welfare, forensic mental health, psychiatric clinic, etc.), up to 19% of children (12), 39% of adolescents (11), and 55% of adults (13) with FASD have been reported to experience some form of suicidality (14). Preliminary work has also been conducted exploring the contextual factors that may be related to suicidality in this population. Co-occurring mental health and substance use needs, neurocognitive and behavioral challenges, problems with independence, housing, employment, and financial stability, and interpersonal stressors, including trauma, are often experienced by individuals with FASD who report suicidality (10, 15). Importantly, most previous research on suicidality in FASD has occurred in clinical settings with suicidality often being a tangential focus (15). Very few studies have incorporated the views of caregivers or explored the deeper lived experiences of suicidality among individuals with FASD and their families.

### Caregivers' lived experiences with suicidality

Caring for an individual experiencing suicidality takes a heavy toll on the whole family system and can negatively impact both the physical and psychological wellbeing of the caregivers (16). In non-FASD populations, suicidality of a family member can significantly influence caregiver mental health, family functioning, and overall wellbeing (17, 18), and can contribute to caregiver burden, pressure, powerlessness, secrecy, shame, and guilt (16, 19). Caregivers of individuals experiencing suicidality have described living in a hypervigilant state, required to ensure the safety of their family member, and experiencing additional stress and fear related to what they may come home to (16). Parents of children who engage in self-harm often develop feelings of helplessness and doubts about their abilities to cope as a parent.

Families raising children with disabilities experience higher levels of adversity including adverse family experiences (20). In general, caregivers of individuals with FASD report exceptionally high levels of concerns and stress (21–24) as well as numerous barriers to adequate services and supports (25). Parents of children and youth with FASD are often aware of and attuned to changes in mood and depressive symptoms which can be extremely taxing and demanding. In a recent study, parental monitoring was identified as playing a critical role in lowering the risk of suicide at times when depression increased for youth (26), and parental monitoring and checking in regularly with children and youth has been shown to increase parental knowledge generally about the wellbeing of their child (26). Research on caregiver experiences of children and youth with FASD suggests that they often face challenges in self-care and the many challenges in caring for children with FASD has an effect on family relationships and contributes to social isolation (27). However, many families of individuals with FASD also show remarkable adaptation and resilience and are able to articulate strengths and values of their families (24, 28, 29).

Given the high rates of suicidality reported among individuals with FASD, and the integral role of caregivers in the prevention of suicidality in other populations (19), further research is needed to better understand the perspectives and experiences of individuals with FASD and their caregivers around suicidality. Given that both social support and depression are important clinical markers of suicidality, these factors are important in prevention and intervention initiatives (26, 30).

### Social-ecological framework of suicide

To account for the complexities of suicide research, practice, and policy, Cramer and Kapusta (31) developed the social-ecological suicide prevention model (SESPM). The SESPM is a multi-level conceptual framework based on the Centers for Disease Control and Prevention's social-ecological framework for violence prevention (32) and aligning with Bronfenbrenner's ecological systems theory (33). The SESPM incorporates four layers of risk and protective factors including (from macro to micro) the societal, community, relational, and individual influences on suicidality. Societal factors are larger scale issues such as social and cultural norms, policies, and other guiding rules or laws, whereas community level factors are those delineated to a certain region such as neighborhood centers, schools, workplaces, and health care providers. Relational factors are defined by direct person-to-person interaction such as social support, peers, and family, and individual factors relate to personal characteristics such as demographics, attitudes, and health conditions (31). The SESPM is not a suicide theory itself, rather, it provides an organizational framework for understanding and better integrating suicide research, prevention, and intervention (31) and can be applied across theories of suicide.

Given the multi-faceted and complex needs, strengths, and lived experiences of individuals with FASD and their families, the SESPM was chosen as the guiding conceptual framework for the current study. Within the SESPM, Cramer and Kapusta (31) encourage a nuanced examination of risk and protective factors that may vary across specific populations, where certain factors may be more or less relevant for different groups of people. Particularly considering the recognized importance of adopting integrated approaches for supporting individuals with FASD and their families (34, 35), the SESPM offers a useful perspective through which suicidality among individuals with FASD can be more comprehensively understood. Therefore, in this study we utilized the SESPM as a guiding framework to explore the lived experiences of caregivers whose children and youth with FASD experience suicidality, including their perceptions of their child and youth's suicidal experiences.

## Methods

### Participants

This study was part of a larger project examining suicidality among individuals with prenatal alcohol exposure (PAE) and FASD. Participants in the current study were caregivers recruited through social media (e.g., Facebook, Twitter), local FASD service networks, and key contacts (e.g., clinical and research contacts, key FASD stakeholders, representatives of the Canada FASD Research Network's Family Advisory Committee—a representative group of caregivers across the country who are well-connected to other caregivers) who shared recruitment materials on the research team's behalf. All participants were individuals who previously completed an online survey conducted in an earlier phase of the larger project (36). At the time of the earlier survey, respondents (*n* = 23) were invited to participate in follow-up, in-depth qualitative interviews, and five caregivers (22% response rate) agreed. These participants, representing a range of cultural backgrounds, included four adoptive caregivers and one trustee (who was a former mentor who assumed trusteeship when the youth turned 18 years of age). One adoptive caregiver was interviewed twice about two different adopted youth in her care with FASD who both experienced suicidality. According to caregivers, their children and youth with FASD had a mean age of 14.5 years (range 11–22) and were currently experiencing suicidality or had a history of suicidality.

### Qualitative interviews

Between March and June 2021, six comprehensive, semi-structured interviews were conducted. These interviews were designed to better understand caregivers' lived experiences of suicidality among their children and youth with FASD, and to contextualize the spectrum of suicidal thoughts and behaviors among young people with FASD. Based on previous research conducted with families of children with FASD and informed by biopsychosocial conceptualizations of suicidality among individuals with FASD, 17 open-ended questions were developed specifically for this study, with follow-up prompts if required. The interview was organized into different sections which included: general introductory questions and rapport building, which included check in questions about how things have been going for the child and the family lately; suicidal thoughts and behaviors; non-suicidal self-injury behaviors; help-seeking behaviors and experiences following suicidality; their child's social experiences and friendships; feelings of hopelessness and depression; feelings of belonging, connectedness, and acceptance; and broader impacts on the family unit. Interviewers also questioned participants about things that have had a positive impact on their child's emotional wellbeing to identify protective factors and ways in which strengths, resilience, and health may be fostered for individuals with FASD. Please see Table 1 for an example of interview questions in each section.

**Table 1:** Example interview guide questions.

**Interview guide sections and topics**	**Example questions**
General introductory questions and rapport building	*Tell me about your child. What are they like? What are they great at?* *How has your family been coping in light of the COVID-19 pandemic?*
Suicidal thoughts and behaviors	*Can you explain what aspects of your child's life have a positive impact on their mental health and emotional wellbeing that make it worth living?* *Has your child ever mentioned thoughts of harming themselves (i.e., an idea-like voice inside their head about ending one's own life or intentionally harming themselves with the intent to die)? Can you describe what your child mentioned?*
Non-suicidal self-injury behaviors	*Would you say these suicidal behaviors are different than non-suicidal self-injury behaviors? How do you feel they are different? Or the same?*
Help-seeking behaviors and experiences following suicidality	*What happened after your child expressed suicidal thoughts and/or engaged in suicidal behaviors?* *Did you seek someone else for help? What did this involve?*
Child's social experiences and friendships	*How would you describe your child's social experiences and friendships? Do you think their social experiences impact their suicidal thoughts or behaviors in any way? If so, in what way?*
Feelings of hopelessness and depression	*Has your child expressed feelings of hopelessness or depression? Do you think these feelings impact their suicidal thoughts or behaviors in any way? If so, in what way?*
Feelings of belonging, connectedness, and acceptance	*Has your child expressed feelings of belonging, connectedness, or acceptance? Do you think these feelings impact their suicidal thoughts or behaviors in any way? If so, in what ways?*
Broader impacts on the family unit	*How have your child's suicidal thoughts and behaviors impacted the people around them? For example, have they impacted your own life? The lives of other family members? The larger community?*

Interviews were conducted by two members of the research team, a postdoctoral research fellow and Registered Psychologist (KT) and a graduate student (SH), with oversight from the project's co-principal investigators (DB, KH, and CM). Interviews took place over a secure video conferencing software and lasted between 2 and 3 h. Given the sensitive and emotional nature of the topic, interviewers checked in regarding the family's wellbeing, current supports and services, and current safety plan to manage their child's suicidal thoughts and behaviors on an ongoing basis. Ethical approval for this study was granted by the Conjoint Faculties Research Ethics Board (CFREB) at the University of Calgary (REB20-0428). Informed consent was obtained both *via* a digital consent form and by verbal consent prior to the interview.

### Data analysis

Data analysis in this study was conducted following the principles and practices of interpretative phenomenological analysis (IPA) (37, 38) which aims to get “as close as possible” to the lived experience of participants regarding a particular phenomenon. Using IPA within the current study allowed us to glean insight into the perspectives and experiences of caregivers raising children and youth with FASD who are suicidal or have experienced suicidality. For example, we as researchers gained insight into the emotions surrounding caregivers' experiences, their understanding and meaning-making process regarding the challenges of navigating their youth's suicidality, and our understanding of our participants' meaning-making process (38).

All interviews in this study were digitally recorded and transcribed verbatim. Data analysis was conducted primarily by two authors (KH and MB) who first reviewed all six transcripts several times independently to acquaint themselves with the content of the interviews. Initial notes and comments were made throughout the transcripts (e.g., notations in the margins of the document, highlighting of key passages, and content, etc.). After making initial notes and remarks on the transcripts, the same authors then re-read the interviews multiple times to transform initial thoughts and ideas into more specific preliminary themes and phrases. Data were further reduced by establishing connections between preliminary themes and organizing them thematically. Themes were given descriptive labels that communicated the nature of the theme, using direct quotations from the interviews. Throughout the data analysis process, KH and MB met several times to discuss their thoughts on the interviews, their generated preliminary themes, and to work collaboratively to categorize and refine their initial themes into more fully realized and finalized themes. These on-going discussions provided space for sharing our individual development and construction of the key findings based on each researcher's understanding of the data (e.g., as informed by our expertise in FASD and suicidality, respectively). These meetings allowed for differing understandings or constructions of the key findings to be discussed and debated, leading to an eventual shared understanding and agreement on the final themes generated.

In line with the philosophical underpinnings of IPA, our analysis was conducted first on a case-by-case basis to understand each individual's experience before comparing experiences across our sample or considering them in relation to our conceptual framework (38). Once final themes were determined, we further considered our themes in line with our conceptual framework. All authors iteratively discussed the final themes and our final organization and write-up. As described above, once our final themes were generated using IPA and agreed upon as a group, these themes were then deductively mapped onto Cramer and Kapusta's (31) SESPM. Organized around the four levels of the SESPM, our results were synthesized into a composite vignette that depicted and illustrated the multi-level factors related to suicidality among children and youth with FASD at the individual, relational, community, and societal levels.

Drawing on the work of scholars in other fields who have used vignettes as a method to present research findings (39, 40), we used a composite vignette to depict a mix of experiences that are fused together into a single all-encompassing narrative (39, 41), organized around the SESPM framework. Given the depth and sensitivity of details shared with the research team during the data collection process, the research team recognized that there was a potential risk for our participants to be identified if individual level quotes were presented that were attributed to a particular participant, even with de-identified information and the use of a pseudonym. It was of the utmost importance to the research team to present our findings in a way that authentically expressed the experiences of our participants and their children and youth, while also protecting their anonymity. The research team strongly felt that to present our findings in an alternative way, such as themes with corresponding quotations, would require the diluting of meaning of the complex stories that our participants shared and would in fact take away the voices of our participants who had shared the sensitive details about their experiences with us, making clear the urgency for these conversations. Therefore, to protect the anonymity and confidentiality of our participants, and to share our findings in an authentic and impactful way that provides a voice for our participants without adding to their vulnerability, a composite vignette was written to meld together stories, experiences, and voices across all six interviews into one synthesized narrative.

As described by Schinke et al. (40), composite vignettes enable researchers to bring together various elements of participants' stories that weave together a more powerful and comprehensive shared account of the phenomena at hand. When constructing the vignette, a narrative outline was first created using the final themes generated and following the structure of the SESPM. KH reviewed the data under each theme, extracting key words, phrases, quotes, and stories that best represented each theme in relation to the four levels of the SESPM. Data extracts were organized, re-organized, and pieced together to establish a compelling story (40) with consideration of the challenges, strengths, and protective factors of children and youth with FASD and their families. In the final composite vignette, direct quotations from the data generated as part of each theme were kept and used to preserve the participants' voices and lived experiences. All authors reviewed and revised the final vignette presented here. We note for the reader that the composite vignette is a particularly sensitive piece of writing, given its melding of many vulnerable and traumatic experiences for individuals with FASD and their families.

## Results

The composite vignette described below reveals the narratives of families living with and caring for children and youth with FASD who have experienced suicidality, in relation to individual, relational, community, and societal level contextual and protective factors. The vignette is personal and intense, at times conveying the heaviness of caring for a young person who has been actively suicidal. Family voices express the trauma and grief that they have experienced. The vignette directly reflects family voices and social location as shared with the researchers and the myriad of experiences of families in terms of both strengths and vulnerabilities. The risk of suicide is real in these families, but this is balanced by all that families do for their children, to make them feel safe, to access supports, and to protect them at all costs. The families involved in this research were exceptional in their efforts to support and care for their children and youth during the times they experienced suicidal thoughts and behaviors.

In level one, we explore the complex individual-level factors that caregivers perceived to be related to suicidality among children and youth with FASD. These included socio-demographic characteristics, co-occurring health conditions, substance use, early life trauma, and familial conflict. Participants also spoke of the individual protective factors, such as the pursuit of personal interests, physical activity, and time in nature which help mitigate risk. In level two, we identify the relational factors that may be associated with suicidality among children and youth with FASD, including feelings of belonging (or lack of belonging), social disconnection and bullying, and the influence of peer groups. Participants also discussed the impacts of suicidality on the caregiver and the whole family unit, including specific stressors, family dynamics, and the coping strategies. Level three encompasses the community level influences of suicidality, centering regions or settings, such as neighborhoods, schools, workplaces, and interactions with health care systems. In level three, we identified a particular emphasis on the consequences of late or non-existent access to mental health supports and services for children and youth with FASD and their families. Participants also addressed the helpful interactions they have had with service providers and more informal support networks which have improved their wellbeing as well as the health and safety of the child or youth. Finally, in level four we identify the societal level factors related to suicidality which emphasize larger scale issues including stigma, geographic region (e.g., urban vs. rural settings), and the impacts of the COVID-19 pandemic on family experiences related to suicidality.

### Level one: Individual level factors

My child's suicidality emerged at a really young age. I think the thoughts really started when they were about 5 years old. They have expressed being depressed since they were about 7 years old and that feeling has never really left them. At 7 is also when they told us that they were a boy, and we kind of took things slowly. And then at 13 when they started puberty and their menses, it really hit that they're a boy.

Their life has been really, really hard. They came from a very traumatic background before they came into our lives. They have a number of siblings, all of them who are on the spectrum. Some diagnosed with FASD, some undiagnosed, but there's no doubt in my mind. Their mother passed away when they were a child, and their father has been in and out of their life. Their father has bailed on them more times than I'd like to count. They have diagnoses of separation anxiety, oppositional defiant disorder, borderline personality disorder, attention deficit hyperactivity disorder, bipolar disorder, intermittent explosive disorder, and learning disabilities, all on top of the FASD. They have also struggled with alcohol and drug use, especially during their early teenage years. They were sexually assaulted when they were a child by an extended family member from their birth family. Their siblings would call on them and ask for money, or tell them to get back into contact with their birth father, and so that always ended up being really traumatizing and triggering. They didn't have that kind of loving support that they deserved and should have had, and so it was during those times that they ended up using alcohol and became addicted to crystal meth. So I think all of these complex life experiences led to their suicidal thoughts and behaviors.

The suicide talk for them at this point occurs daily. They will often say things like “Why am I here, nobody loves me, I just need to die”. Honestly, a lot of “I'm just gonna go kill myself, I'm just gonna run away, I'm just gonna die out in a field somewhere.” Sometimes the suicidal thoughts come from anger at themself or anger at us. They'll say things like “I hate myself, I can't stop my brain from doing these things, I hate you, I don't want to be part of this family, I wish I was never here.” It's really emotional, just so intensely emotional. They're such a big ball of love, but they also have a lot of really big emotions. The thoughts and talk have been really scary and really concerning for us lately, especially because of their history of cutting and because of their previous suicide attempt. We are hypervigilant at all times. It's hard because we live on a farm and we have guns out here. They're locked away like they're supposed to be, we follow the rules, but being out on a farm… When these thoughts come up we make sure they stay with us, like you are not leaving my side, you're sleeping in my bed! Because I need to know what their every move is, for at least until I know that they are safe. I have all of my sharp things locked up and out of sight too, so I also make it really hard for them to get access to anything which they can use to harm themselves.

We work really hard to refocus them and distract from the thoughts when we see the volatile emotional swings. We worry that they are getting closer to the thoughts that would actually… lead to the end of their life, but we try to focus on the fact that it's very much in the moment. Right now I don't think they have the process to think of how it will actually work to kill themself. They can't connect that their act would not cause any serious harm… So I don't know if they would follow through now, but I am concerned about what will happen when it all starts to catch up to them as they get older.

For now, we really just try to focus on their strengths and to try to get them out doing things that they enjoy. I think they really dive into their art, and they like gaming, so we try to help with that so that they don't get stuck in the thoughts. They really like exploring new things as well, and getting outside. The physical movement, like running and climbing and bike riding is really good for them. The biggest positive and help is if they get outside. If they are stuck in the house too long their mental health state just deteriorates.

### Level two: Relational level factors

For my child it is all relationship based. The relationships with peers and friends, romantic partners, and family members have such a big impact. The most recent challenge has definitely been related to school. Because of overcrowding in the town where they went to school, they were taken out of school and put in the high school. That was really hard at the time because they were not really 13 or 14 mentally, their social or emotional age was maybe 9 or 10. They have experienced so much bullying at school during their life and I think a lot of that is related to their personal characteristics—their identity, their appearance, and also their developmental capabilities. They've had teachers, principals, students, even community members bully them. The bullying at school lately though has really been a problem. One day they came home from school and told us that a bully at school was making transphobic and homophobic attacks and using pandemic rules to isolate them from their friends. The rule at school was that only two kids per cohort were allowed to sit in the lunch group at a table, so this bully would manipulate the rules and use these rules to isolate them from the rest of the peer group.

In elementary school they were also teased remorselessly at school. They were quite a bit bigger when they were younger, and kids used to tease them for being fat. One time two boys put sticky notes on their back saying “I'm fat and I'm stupid” and they walked around school like that. Other kids used to ridicule them and then be like “Come play soccer!” Then the kids would kick balls at them. I would tell them that the kids were being mean, but they would say “They're my friends.” And I would have to say, “No sweetheart, they're not, they're being mean to you.” So, for a long time it really was teaching them and reminding them what a friend looks like and what somebody who is not a friend looks like. I am so thankful that they have managed to find a small group of friends that are really good to them now.

Their peer group is seriously the greatest thing that could have ever happened to them. For a long time, they didn't really have friends, and didn't know how to have friends or make friends, so this strong peer group that they have developed has really been amazing. I am particularly grateful for their closest friend from this peer group who intervened when they were worried about my child. A few months ago, my child said to this friend that they didn't want to be here anymore and they kind of started saying goodbye to their friend. This friend went straight to their mom and showed their mom the texts from my child, and within a minute this mother called me and told me what was going on. So probably within 5 min of that text message of my child saying goodbye to their friend, my husband and I were downstairs intervening. We didn't take their phone away, because clearly that was the tool that saved their life and I'm not going to punish them for saying they need help. So really this peer group has been so important for them in a lot of different ways.

But, while I am so grateful for this peer group right now, it's honestly my next big fear and that's what I'm trying to prepare them for. I have talked to them about their differences… that differences are okay, but their peers may grow out of these friendships and that's okay. I've also tried to prepare them for the next phase in life that is happening… Because like I said, these friends are great and I love them dearly, but be prepared that they're going to want boyfriends and girlfriends and partners, and maybe they want to go do something else. And they'll always care about you, but they may not always be there and that's okay. I feel like they are maybe starting to understand this dynamic though because they were kind of in a relationship with a girl from an FASD support group for teens. The romantic relationship piece is a whole new terrain for us at this point as our child gets older. I think one thing that would really drive them over the edge is if their relationship with their girlfriend was to end. I worry that when they have fights or disagreements with their girlfriend that it is contributing to the daily “suicide talk”.

So certainly, there is a lot going on in our lives. For us as a personal family, I think we are really on edge. When they are having a good day, it's good. But when they are even a little bit off it's really hard, and it's especially hard for us to sleep. It's like we are on watch all the time if they sneak out of their room. It's on watch for everything. If my husband takes a knife out to cut a bagel, it's like immediately washing it and putting it back in the safe. We're definitely on high alert. Beyond our immediate family unit, it has also led to challenges with our extended family. Other family members are just really nervous to be around them.

My husband also travels quite a bit for work and so when my husband is home, he has a hard time dealing with them sometimes because of the FASD and how encompassing it is. My child often feels that their father doesn't love them because of their FASD. It's not that he can't wrap his head around the FASD, and certainly he loves them, but it's … complicated. They can be very difficult and there's tension. My husband really is not dealing with it well at this moment in time. He gets angry with them. Sometimes they will yell and scream at us and sometimes that is directed at my husband in particular. I'm very up front with it. I will say to my child, “You're being disrespectful, you're being unkind, you're being selfish.” I use all of those negative things I shouldn't say and it makes them feel bad, right? Which I know and I feel bad about, and we talk about it a lot. My husband and I are doing more FASD training stuff. It's like I *know* this stuff, why can't I just stop myself and leave them alone and let them yell and scream when they need to and just not say anything? But, you know, they trigger me too sometimes. So as for me, I am always stressed. I have some health issues because of all of these stressors. I have post-traumatic stress disorder from it. I just do my best to get through every day. There's nothing else I can do, I just do my best every day and get up and just keep going.

Despite the stressors, one thing we really try hard to do is to always go over to them, always give them hugs and kisses, and tell them how much we love them. It can be hard sometimes, but we always want them to know how much and how deeply we care about them. They do have an older sister, our biological daughter, who is almost 30 now, but hanging out with her can be really good. They are super attached to her. So that's been really good for them to hang out with her. If we can try and get them distracted, our eldest daughter is usually really good if she's around. She can usually do something to get them distracted. I have no idea what, but she just has a gift with them that the rest of us don't.

### Level three: Community level factors

Overall, we've had very negative experiences seeking help which has been so unfortunate because we actually moved just so that my child could get support. We used to live up in the north and my child saw that a number of the other younger children were cutting. That was how they first got the idea in their head when they saw others doing that, and after that they cut for 2 ½ years. It was a big thing in that community.

When it comes to medical professionals, that has been a real challenge. I'll do whatever it takes to keep my child safe, but I have a lot of trepidation about the medical system. I have a lot of fear and a lot of distrust of the medical system, so I have to say it would be an honest last resort to take them to a hospital. I would really try everything in my power to keep them safe before it got to that point. We're having a hard time getting them in to see a psychiatrist right now, but I definitely want to get them help. I just want to get them general therapy help for sure. They would do really well with therapy one-on-one and having to actually *go* to therapy. We were trying virtual therapy earlier this year but this Zoom therapy just does not work for them. So we had to stop that because every time it was a fight and they would just yell “no, no, no, I'm not doing that!”. I can only push so much before I shove them off a cliff and they won't talk to me about anything, so I'm really trying to balance respecting their boundaries vs. pushing them still a bit.

We do also have a respite worker, but the respite worker is terrified of them. Even if we could get the respite support going, access has also been a problem. It's been very short, like 8 h a month. That's not enough for FASD. When the worker comes, it almost triggers them more because it's so little. They need a constant routine and from 1 week to the next is huge in their brain because of their memory challenges. So when there is no consistency, that throws a wrench into it. It makes it worse and then it triggers them which can make them very angry and then we go down a whole rabbit hole… Support is really something that we have been thinking about a lot lately because we've been thinking about the big picture, not just the everyday day-to-day. I hope that eventually we will get something consistent and helpful for them because their situation and their challenges are lifelong, and we need to set them up for the lifelong, not just the day-to-day.

Along the way there have at least been a few professionals who have been helpful. My child had a few amazing teachers along the way who were very, very good. They also had a really good advocate at the school who was a coach. But really, I think we need more people who actually know about FASD and know how to help. There is such a lack of support for FASD and it is so encompassing. It's not just one issue—it's their mind, it's their body, it's everything—and trying to find somebody that can address the encompassing nature of FASD is impossible.

The one thing lately that has really been helpful is that I'm starting to get better connected, or at least trying to get connected, again with some more support groups. I do have a group of moms that I connect with sometimes too—there are six of us and we all have children with FASD. Each child has different levels of functioning, but all of the youth are relatively close in age, so I draw on their support. They're also all professionals of different levels in their own right. I'm also really good at making friends with other parents in general because I've just learned that it's the only way I can keep my child safe. I'm a very loud and outspoken advocate for my child. For example, because of all of the bullying at school, I made the school have a safety plan in place before I would send them back to school and I was not very pleasant about it. I was pretty harsh with the school because they are very aware of their disability, they are very aware of their struggles, and I just felt like they were not participating in keeping them safe. I have really tried to build this village around them to make sure that they are safe, supported, and understood.

### Level four: Societal level factors

The pandemic has definitely exacerbated things for our family. These emotional outbursts, the daily suicide talk… Since the pandemic, it's definitely increased. “You want me dead, you don't want me alive, I want to be dead”, those types of things. But I think all kids are feeling that kind of languish right now, you know? That feeling like you're trapped and all you can do is walk the same circle, over and over again. One of our go-to activities as a family before the pandemic was swimming. We would swim at least twice a week and that is their one sense that just helps them get released. A bathtub is not the same as a swimming pool. So not having access to a swimming pool was very detrimental to their mental health. The lockdown definitely made things worse, but I'm also kind of grateful that it happened because it opened up the conversation for us. It's kind of a double-edged sword. I'm grateful and I'm hateful, it's kind of both. But that's the joy of living in this reality I guess! It's like that Disney Pixar movie, ***Inside out***.

You can have multiple feelings at the same time, you can be happy and sad and mad and hateful all in the same moment and that's what I was feeling.

Beyond the pandemic, the other thing that I worry a lot about is how the world will continue to respond to and treat our child. Our child is also a member of a racialized group, so we talk a lot about what it is like having a dark dad and a white mom and the risks those carry in the world. We're not ones to go and lock ourselves on a door or anything, but we're activists. We carry an activist heart. We're always striving to improve the community and it's important to dispel a lot of misinformation that people have about BIPOC. I'm really trying to break down stereotypes and stigmas and advocate for my child, both when it comes to fighting stereotypes… and also the stigma that exists toward people with FASD. It's not their fault. I'm really trying not to put blame on my child for their actions, because it's brain damage, and I just wish people had a better understanding of FASD.

## Discussion

In this study, we explored caregivers' lived experience related to FASD and suicidality. Participants spoke to the complex and intersecting individual, relational, community, and societal level factors associated with suicidality among their children and youth with FASD. Until recently, research on suicidality among individuals with FASD has been limited (10, 12, 14, 15, 42–44), and few researchers have explicitly sought to examine suicidality among individuals with FASD as the main purpose of their study (13, 14, 45, 46). The results of this study offer crucial insight into the range and contexts of suicidality in FASD, as well as the potential impacts of suicidality and associated support needs of individuals with FASD and their families.

### Key findings regarding suicidality among children and youth with FASD

One of the key purposes of this study was to explore caregiver perceptions about factors associated with suicidality among their children and youth with FASD. As described by our participants, these factors spanned all four levels of the SESPM and included complex trauma, stress response, co-occurring mental health and substance use challenges, experiences of stigmatization and racism, bullying, social isolation and marginalization, and lack of societal awareness, understanding, or compassion regarding FASD. Specifically, caregivers described how the interplay of these factors were all relevant and significant factors that resulted in a culmination of challenges for their children and youth that led to suicidality (31). Caregivers spoke to the layered complexity of these experiences considering the COVID-19 pandemic and how the pandemic as a societal level concern further heightened their children's mental health concerns suicidality. The complex and unique experiences and vulnerabilities of individuals with FASD have been well-documented (6, 7, 9) and the findings of this study provide further evidence of the significant challenges individuals with FASD and their families experience in their daily lives. Caregivers spoke to the brain and body-based challenges of their children and youth (e.g., impulsivity, emotional reactivity, memory challenges), as well as how these challenges are often exacerbated based on their child's interactions in various social situations or circumstances (e.g., in school or among peers, with health care or mental health providers) which further heightened risk of suicidal thoughts and behaviors.

Our findings related to contextual factors associated with suicidality in FASD align with previously identified factors, including co-occurring mental health conditions and other neurocognitive and behavioral challenges (10, 13, 44, 45, 47). With consideration of the SESPM conceptual framework, it is particularly notable that these factors overlap and intersect with *all* of the factors that Cramer and Kapusta (31) have identified as being most strongly associated with suicide risk across the multiple levels: mental health diagnoses or symptoms such as depression and bipolar disorder; personality disorders such as borderline personality disorder; substance use/abuse; alcohol use/abuse; prior suicide attempt; current suicidal thinking; access to or the presence of lethal means; hopelessness; and feelings of burdensomeness. The overwhelming prevalence of these risk factors among individuals with FASD highlights a critical need to further develop and enhance targeted protective factors to further support individuals with FASD, including strengthened social support, psychological coping skills, hopefulness and positive future orientation, and identifying additional reasons for living (31).

Several findings in this study are especially notable given recent emerging evidence related to suicidality in non-FASD populations. For example, caregivers identified that their children's individual level mental health challenges, gender identity, and developmental capabilities were related to their youth's suicidality, particularly as these factors intersected with relational level experiences of bullying and victimization from peers and others in the community because of their perceived differences. Recent research with LGBTQ2S+ youth who died by suicide revealed that many were found to be bullied before their death. In reviewing the death records of youth who died by suicide, LBGTQ2S+ youth were almost five times more likely to have bullying documented in their death records compared to the records of non-LGBTQ2S+ youth (48). Younger children in this same study were also identified as being at greater risk, with bullying being reported for two-thirds of youth aged 10–13 years before their death (48).

These findings are highly concerning in conjunction with recent evidence about the mental health related impacts of the COVID-19 pandemic on children and youth's mental health, including among LGBTQ2S+ youth. Rico et al. (49) revealed that children and youth's mental health was highly impacted during the pandemic as a result of social isolation, with nearly half of the youth sampled reporting feeling persistent sadness or hopelessness during the pandemic, and almost half of LGBTQ2S+ youth reporting contemplating suicide during the pandemic. Very little is currently known about the experiences of individuals with FASD who are transgender (50), but the findings of this study considered with recent literature on youth in general speak to the potentially heightened risk for youth with FASD who are highly marginalized in addition to the layered complexity and adversity that they already experience in their daily lives. Caregivers in this study also identified cognitive distortions by individuals with FASD that align with a desire to escape from their sense of burdensomeness arising from thwarted belongingness, which will require a special targeted education and support (51). Further research exploring the individual level factors for suicidality among individuals with FASD, including sex, gender, and other sociodemographic factors, including the social determinants of health, is warranted.

Another concerning finding was the young age at which suicidality first emerged among children and youth with FASD, which is consistent with other research on developmental disabilities and suicidality (10, 36, 52). Caregivers in the current study reported that their child had experienced suicidality as young as 5 years of age. Although rates of suicidality may be highest among adolescents, in one study, researchers reported that 12% of school-aged children with PAE in Canada experienced past or present suicidality (10).

Caregivers reported that their children and youth with FASD engaged in daily or almost daily “suicide talk.” Given the reported cognitive challenges associated with FASD (53, 54), it is possible that some children and youth with FASD may not fully appreciate or comprehend the meaning of their statements about wanting to die or not wanting to be alive. Furthermore, some children or youth with FASD may lack the skills to cope with distressing emotions or adverse events, resulting in volatility, impulsivity, or reactivity to “in the moment” situations, which may lead them to engage in “suicide talk” to express their desire to escape these hard emotions. As caregivers described, “suicide talk” often emerged as a result of a particular antecedent, often a relational issue, and moved quickly to outward expressions of suicidal thoughts (36). Impulsivity is a widely-reported challenge in FASD (55) and has been identified as a factor associated with an increased risk for suicidality in non-FASD populations (56, 57), warranting further investigation among individuals with FASD in the context of suicidality. Notably, caregivers in this study described different strategies and in-the-moment tactics that may be helpful to distract or dissuade someone from suicidal thoughts or expression. Distraction and redirection appear to be critical tools for caregivers of children and youth with FASD and this phenomenon warrants further research to understand how suicidality, impulsivity, and distraction/redirection may interact differently in the FASD population.

It is also evident from the voices of caregivers that relational factors, including family and interpersonal stressors and other experiences of trauma, were highly relevant in the context of their youth's suicidality, again aligning with previous research findings (13, 36, 45). Bullying was identified as one of the most significant relational level factors for youth in this study. Although individuals with FASD are often reported to be highly social and inclined toward human connection (29), they may also struggle with desirable social skills, understanding social expectations, making friends, and picking up on cues associated with bullying (27, 58, 59). Nonetheless, although caregivers in this study reported that their children and youth experienced social isolation and marginalization at times, some youth with FASD also had strong and reliable peer groups of genuine friends. Social support is a critically important protective factor for suicidality in youth with and without FASD (31) and facilitating meaningful social connections and relationships should be of the utmost importance for service providers and others supporting individuals with FASD.

### Key findings regarding caregivers' lived experience of FASD and suicidality

Beyond the insights caregivers provided about the contextual factors pertaining to suicidality among their children and youth, this study also allowed for insight into the experiences of caregivers who are also grappling in their own ways with their youth's suicidality. Specifically, the results of this study provide the first glimpses of the distressing and disturbing experiences of caregivers and align with previous research emphasizing the ways in which suicide-related stressors can interfere with caregivers' relations and quality of life (60). The disproportionate rates of suicidal ideation, attempts, and completion among those with PAE and FASD (13, 14) elevate risk in their caregivers above and beyond the normal population risk. Caregivers of individuals with FASD already experience disproportionately high levels of caregiver stress (21, 22, 61) and the results of this study provide further contextualization of the heightened stressors caregivers of children and youth with FASD may experience if their children are also experiencing suicidality. As caregivers in this study described, their child's suicidality contributed to familial and relationship challenges within the immediate and extended family unit, caregivers' own post-traumatic stress disorder and adverse childhood experiences, and constant states of hypervigilance to keep their children safe, as has been shown in the non-FASD literature (16). Unfortunately, for many caregivers, accessing FASD-informed supports and services was severely lacking, which aligns with previous findings in the FASD field that caregivers often face numerous barriers to adequate service provision (25).

Despite these profound difficulties, it is also important to acknowledge and emphasize the strengths, resilience, and coping strategies reported by caregivers in this study. For example, having positive outlets such as physical activity or art, and accessing strong social connections, offer important examples of how protective factors can be identified and built upon, no matter how small, to support individuals with FASD and their caregivers. Caregivers described the immense care, love, and respect that they have for their children and did not convey a sense that their children are in any way a burden to them. In fact, all caregivers described the joy their children brought to their lives and spoke to their strengths and abilities. Suicide theory, assessment, and prevention requires nuanced consideration of both the risk and protective factors and therefore it is imperative to identify both the challenges and strengths of individuals and families when considering suicidality among individuals with FASD (31). There is a clear need for more intentional studies that focus on protective factors, resiliency, and positive coping strategies among children and youth with FASD regarding suicidality.

### Implications for mental health professionals

Mental health professionals and clinicians supporting individuals with FASD have an essential role in both prevention and intervention to reduce the risk of suicidality. It is critical to advocate for school system strategies to eliminate bullying and provide supportive counseling for individuals with FASD on how best to navigate peer relationships. Mental health professionals need to have a basic working knowledge of the mental health problems associated with PAE and FASD and require training on how to intervene with individuals with FASD. Further training with relevance to minimizing suicidality should target the need for consistent guidance, stigma reduction, need for early intervention, and need for support services. Additional support can be gained when professionals play an active role to advance caregivers' understanding of the common risk factors for suicide, the interpretation of suicidal statements, and important steps to take to seek help and intervene (62) if and when their child does express suicidality. Such steps involve expanding the support and health care network around the individual across physical, psychological, spiritual, and emotional domains (63). Caregivers should also be trained in becoming aware of the emotional pain their loved ones with suicidality experience, how to promote positive attitudes, and how best to care for and support their loved ones when suicidal (64). Transitions in care such as a placement move are a crucial point in suicidality that require the support of caregivers (63). The lack of knowledge about FASD and suicidality among mental health professionals builds a compelling case regarding the need for clinical and caregiver support in assessing, managing, and responding to the expression of suicidal intention (65). By adopting a biopsychosocial approach that considers the complex social-ecological factors explored in this study, clinicians can employ advances in managing mental health challenges often experienced by youth with FASD and target specific interventions for suicidality.

As executive function, motor speed function, and global neuropsychological function are associated with suicidal ideation in patients with mental health issues, future research should target the role of caregivers in moderating the effect of PAE related cognitive difficulties in predisposing and perpetuating suicide risk (66). Clinicians should also be alert in recognizing the red flags of FASD in their practice at various service access points and as individuals navigate the health system (65). As caregivers in this study described, current service systems are not well-equipped to support youth with FASD and even if they gained access to care for suicidality, the care was often not appropriately tailored to meet the unique and complex needs of the individual and their family. While mental health professionals can play an instrumental role in supporting individuals with FASD and suicidality, the need also exists for FASD informed mental health professionals to support caregivers who live day-to-day and often intervene daily to the suicidal thoughts and behaviors of their children and youth to have access to supports should crises arise.

### Limitations, strengths, and considerations

Although this study offers some important insights that expand our understanding of suicidality among individuals with FASD and their families, it is not without limitations. The first limitation of this study is the reporting by caregivers on behalf of their youths' experiences. Our understandings and interpretations presented here are therefore limited to the suicidal thoughts and behaviors that could be detected by caregivers, so it is possible that caregivers may not have always recognized risk or known about other instances of suicidal thoughts and behaviors. The views of caregivers presented here may also be shaped and altered by the distressing experiences some described. Furthermore, movements such as *nothing about us without us* (Charlton, 1998) emphasize the voices of the individuals directly impacted and so it is important to note that the views of caregivers presented here may not align with the youths' views of their own experiences. Although caregiver perspectives are an essential part of the complex picture of suicidality among individuals with FASD, future research is needed that centers on the voices of those individuals with FASD with lived experience. A second limitation of our study was the structure of the interview guide that focused predominantly on caregivers reporting about the experiences of their children and youth and not as directly on the way in which caregivers navigate their children's suicidality. Although questions about the caregivers' lived experiences were not explicitly asked, the nuance and the depth of the data collected during the interviews allowed for detailed interpretations of caregivers' lived experiences.

However, a key strength of our study is the use of, and grounding in, the multi-level conceptual framework (31). Use of this comprehensive conceptual model provided an organizational framework for generating knowledge about the risk and protective factors for individuals with FASD and their families across the four social-ecological levels. The use of the SESPM as a guiding flexible framework allowed us to understand and integrate the complex picture of risk and protective factors for suicidality among individuals with FASD and their families, articulating the complexity of life experienced by individuals with FASD.

Finally, during the interviews, and particularly at the end of the interviews when we asked caregivers to share any final thoughts, caregivers spoke a lot about FASD being an understudied and often invisible disability group. Caregivers expressed their immense gratitude that the unique experience of FASD and suicidality was being considered and investigated, which speaks to the novelty and significance of this work and the direct impact this research may have for families experiencing these challenges every day.

## Conclusion

The intent of this research was to provide insight into the experiences of families caring for young people with FASD experiencing suicidality. The creation of a composite vignette offered a unique approach to reflect the voices of caregivers and highlights the intensity of their lived experience. Coupled with previous evidence of the remarkably high rates of suicidality among individuals with FASD, our findings related to the multi-level complexity of suicidality and its impacts on caregivers and families underscores the need for mental health and other professionals to be trained in FASD. It is recognized that family and community can serve as critical protective factors to mitigate the risk of suicidality, being mindful that supports for individuals and families must always be in place to address complex biopsychosocial needs. Caring for children with FASD who experience suicidality places a heavy burden on caregivers who often seek help from systems with little knowledge about FASD and mental health. Advocacy is urgently required to ensure that mental health systems have the knowledge and capacity to support caregivers of children and youth with FASD who need assistance in navigating the complexity of suicidality.

## Data availability statement

The datasets presented in this article are not readily available because participants of this study did not agree for their data to be shared publicly. Requests to access further information about the study should be directed to KH, kelly.harding@canfasd.ca.

## Ethics statement

This study involving human participants was reviewed and approved by the Conjoint Faculties Research Ethics Board (CFREB) at the University of Calgary (REB20-0428). Informed consent was obtained both via a digital consent form and by verbal consent prior to the interview.

## Author contributions

KH, KF, and MB worked together to write the first draft of this manuscript. KT and SH conducted the qualitative interviews reported on in this manuscript. KH and MB conducted the data analysis for this manuscript with input and feedback from KF, CM, and DB. All authors contributed writing to sections of the manuscript and to the conception of this manuscript. All authors read, revised, and approved the submitted version.

## Funding

This research was supported by PolicyWise for Children and Families (research grant 10028277). Publication of this article was supported by the Canada FASD Research Network.

## Conflict of interest

The authors declare that the research was conducted in the absence of any commercial or financial relationships that could be construed as a potential conflict of interest.

## Publisher's note

All claims expressed in this article are solely those of the authors and do not necessarily represent those of their affiliated organizations, or those of the publisher, the editors and the reviewers. Any product that may be evaluated in this article, or claim that may be made by its manufacturer, is not guaranteed or endorsed by the publisher.
